# Testing the Insider Trading Anomaly in FTSE-350

**DOI:** 10.3389/fpsyg.2022.751665

**Published:** 2022-02-15

**Authors:** Jinxia Meng, Leping Huang, Zhou Lu

**Affiliations:** ^1^Jiaxing Vocational and Technical College, Jiaxing, China; ^2^School of Foreign Languages, Shenzhen University, Shenzhen, China; ^3^School of Economics, Tianjin University of Commerce, Tianjin, China

**Keywords:** insider trading, event study, small-firm effect, market efficiency, FTSE-350, D82, G32, M40, G14.

## Abstract

In recent studies, numerous anomalies against the weak and semi-strong-forms of efficient market hypothesis (EMH) have been found insignificant after controlling the small-firm effect. We investigate whether the insider trading anomaly, a major anomaly against the strong-form of EMH, can survive after excluding small firms with a novel data set (FTSE-350) and document several new findings. We find a substantially larger number of insider purchases than sales, while the average volume of insider sales is much higher than the average volume of insider purchases. Echoing recent US studies, we find that insider sales generate more abnormal returns than insider purchases do. We find much lower abnormal returns from insider trading than documented in the literature and the associated trading costs, which suggests that the market efficiency of individual stocks may depend on their sizes, and even the strong-form of EMH holds to a larger extent than previously recognized.

## Introduction

There are three forms of efficient market hypothesis (EMH)—weak-form, semi-strong-form, and strong-form efficiency. The first two forms imply that technical analysis and fundamental analysis should not work, while the strong-form EMH suggests that even insider trading should be unprofitable. Evidence contradicting any of these three forms is generally referred to as a market anomaly, including technical anomalies (e.g., momentum effect), fundamental anomalies (e.g., size effect and value-vs.-growth effect) and the insider trading anomaly. Anomaly literature has become one of the largest strands of literature in Finance, with hundreds of anomalies documented in recent decades.

Are these anomalies really anomalies or just artifacts due to data mining and/or publication biases (e.g., Harvey, 2017)? This question is of essential importance and has attracted increasing attention since the late 1990s. For instance, Fama (1998) documents that many anomalies tend to be less significant or may even disappear when they are measured with value-weights instead of equal-weights. Fama and French (2008) further demonstrate that there are fewer anomalies in big stocks than in small (especially microcap) stocks, which are less liquid and associated higher transaction costs and hence difficult to generate anomalous returns in reality. More importantly, the economic significance of the microcaps is negligible, as they account for only 3% of the total market capitalization of the NYSE-Amex-NASDAQ universe, albeit 60% of the number of stocks. While Harvey et al. (2016) find a large number of false discoveries among 296 anomalies, Hou et al. (2017) re-evaluate 447 anomalies and deem more than half of them artifacts due to overweighing microcap stocks.

However, all of the papers mentioned above focus on the anomalies against the weak-form and semi-strong-form EMH. It is surprising that none of them doubt the credibility of the insider trading anomaly, which is against the strong-form EMH. The magnitude of anomalous returns is negatively correlated with firm size (e.g., Friederich et al., 2002). This is an important task, given the unfairness and unparalleled profitability of insider trading relative to other anomalies. Our paper fills this gap.

Several aspects of this study differentiate it from the vast existing literature on insider trading due to the nature of our research question. First, we deliberately focus on the UK market rather than the US market, as the firm sizes in the UK market are more homogenous than in the US market. Relative to the US market, the research question of UK insider trading is under-researched with a less developed strand of literature.

Second, to wash out potential contamination from the small and microcap firms identified in the literature (Fama, 1998; Fama and French, 2008; Hou et al., 2017), we choose to exclude the FTSE-SmallCap and focus on FTSE-350, which consists of the large-cap FTSE-100 and middle-cap FTSE-250 only. Almost all previous UK insider trading literature uses samples from FTSE-All-Share or London Stock Exchange (e.g., Hillier and Marshall, 2002; Fidrmuc et al., 2006).^1^ According to FTSE Russell Factsheet^2^, FTSE-350 accounts for 96.4% of the net market capitalization of FTSE-All-Share and 55.7% of the number of the stocks, while FTSE-All-Share captures 98% of the UK's market capitalization.

Third, we use a relatively long (10-year) sample, starting from the late 2000s, with daily data. Most existing studies on this topic rely on pre-crisis data and need to be updated. Daily data have two advantages: (1) moderation of event clustering effects from a statistical standpoint (Brown and Warner, 1985); (2) abnormal returns tend to be normally distributed when the sample size is larger than 100 (Campbell and Wesley, 1993).

As regards the methodology, we follow the mainstream literature (e.g., Brown and Warner, 1985) to utilize a standard event-study methodology. We consider both the parametric standardized cross-sectional test suggested by Boehmer et al. (1991) and the non-parametric rank test suggested by Corrado (1989) to address the well-known statistical difficulties inherent in event-based methodologies, such as outliers, asymmetry in cross-sectional excess-returns distributions, and event-date excess-returns variance increases.

We find much lower abnormal returns from insider trading than documented in the literature and the associated trading costs, which suggests that the market efficiency of individual stocks may depend on their sizes from a non-US perspective. Although existing literature describes the small-firm effect, we demonstrate that because the small-firm effect is so large, even the strong-form of EMH holds to a greater extent than previously recognized, after excluding the small firms.

The remainder of the paper is organized as follows: Section Methodology and Data discusses the methodology and data. Section Empirical Results: Short-Term Cumulative Abnormal Returns (CAR) presents the main empirical results. Section Robustness provides a battery of robustness checks. Section Concluding Remarks concludes the study.

## Methodology and Data

### Methodology

#### Cumulative Abnormal Returns (CAR)

To evaluate the abnormal returns from insider trading, we use the standard event-study methodology (Brown and Warner, 1985, p. 7, 9). Denoting event time as *t*, assigning the event day designation *t*_0_, we define the event window (from *t*_*y*_ to *t*_*z*_) from *t*_−20_ to *t*_20_. Approximating expected returns through a simple market model, we assume the abnormal return on security *i*, observed over day *t*, to be defined as:


(1) $$\begin{array}{r} AR_{i,t}=\;R_{i,t}-\;\alpha_{i}-\;\beta_{i}R_{m,t}, \end{array}$$

where *R*_*i, t*_ denotes the observed daily return of security and *i* and *R*_*m, t*_ the market return.

We estimate the market parameters β_*i*_ and α_*i*_ through OLS regression, taking the FTSE-350 index as an effective proxy. Applying a 240-day pre-event estimation window (*t*_*x*_ to *t*_*y*−1_), we regress, for each individual event, *R*_*i, t*_ = α_*i*_+ β_*i*_*R*_*m, t*_ across the period *t*_−240_ to *t*_−21_. Cumulating the abnormal return (AR) to all events on a day-by-day basis across the relevant event window (*t*_*y*_ to *t*_*z*_), yields the below CAR measure where *N* is equal to the number of distinct events:


(2) $$\begin{array}{r} CAR\left(t_{y},t_{z}\right)=\;\sum_{t=t_{y}}^{t_{z}}\left(\frac{1}{N}\;\sum_{i=1}^{N}AR_{i,t}\right). \end{array}$$

#### Parametric Standardized Cross-Sectional Test

We standardize the ARs to prevent the possible distortion effects from outliers by using the parametric standard cross-sectional test statistic (*t-BMP*) of Boehmer et al. (1991). Dividing by the estimated standard deviation (*S*_*i, t*_) yields standardized AR: $SR_{i,t}=\frac{AR_{i,t}}{S_{i,t}}=\;\frac{\;AR_{i,t}}{{\hat{\sigma }}_{i}\sqrt{1+\;\frac{1}{L_{1}}\;+\;\frac{{\left(R_{m,t}-\;\bar{R_{m}}\right)}^{2}}{\sum_{t=\;t_{x}}^{t_{y}}{\left(R_{m,t}-\;{\bar{R}}_{m}\right)}^{2}}}}$, where *L*_1_ and ${\hat{\sigma }}_{i}$ denote the number of trading days and the variance of ARs of security *i* within the estimation window (*t*_*x*_ to *t*_*y*_), respectively.

The test statistic is $\frac{\frac{1}{N}\sum_{i=1}^{N}SR_{i,t}}{\sqrt{\left[\frac{1}{N\left(N-1\right)}\sum_{i=1}^{N}{\left(SR_{i,t}-\sum_{i=1}^{N}\frac{SR_{i,t}}{N}\right)}^{2}\right]}}$, and the multi-day version is.


(3) $$t--BMP=\frac{\sum_{t=t_{y}}^{t_{z}}{\bar{SR}}_{t}}{\sqrt{\sum_{t=t_{y}}^{t_{z}}{\hat{\sigma }}^{2}({\bar{SR}}_{t})}}.$$

#### Non-parametric Rank Test

The non-parametric rank test proposed by Corrado (1989) provides enhanced power regarding thin trading and misspecifications such as asymmetry in cross-sectional excess-returns distributions and event-date excess-returns variance increases (Campbell and Wesley, 1993). It sorts and ranks ARs across both the estimation and event windows, i.e., *k*_*i, t*_ = *rank* (*AR*_*i, t*_) for *t* = *t*_*x*_, …, *t*_*z*_ with the rank statistic $\frac{\frac{1}{N}\sum_{i=1}^{N}\left(k_{i,t}-E\left(k_{i}\right)\right)}{ŝ\left(k\right)}$, where *L*_2_ denotes the number of trading days over the event windows (*t*_*y*_ to *t*_*z*_) and *E*(*k*_*i*_) denotes the expected rank of security *i*, equivalent to $\frac{L_{1}+L_{2}+1}{2}$. ŝ(*k*) denotes the estimated standard deviation of the mean portfolio AR rank over estimation window as given by $ŝ\left(k\right)=\;\sqrt{{\frac{1}{L_{1}+L_{2}}\;\sum_{t=t_{x}}^{t_{y}}\left(\frac{1}{N}\sum_{i=1}^{N}\left(k_{i,t}-E\left(k_{i}\right)\right)\right)}^{2}}\;$, and the multi-day version of the test statistic is:


(4) $$\begin{array}{r} t-\text{Corrado}=\frac{\sum_{t=t_{y}}^{t_{z}}{\bar{k}}_{t}}{\sqrt{\sum_{t=t_{y}}^{t_{z}}{ŝ}^{2}\left({\bar{k}}_{t}\right)}}. \end{array}$$

## Data Description and Preliminary Analysis

Our main data source is the Morningstar Premium Director Share Dealings Database, which provides extensive insider trading information. We obtained closing prices in daily frequency and associated stock ratios from DataStream. All prices are adjusted for stock splits, dividends and related corporate events. Due to data availability, our sample spans the period 1 January 2005 to 31 June 2015, without concentration in any firm, industry, or sub-period. After excluding transactions relating to the exercise of warrants, options, preference shares, and other non-ordinary equities, as well as sales after the exercise of options, our sample consists of 46,318 transactions. Of these, 38,924 transactions (with an average market value of £96,423) are purchases and 7,394 transactions (averaging £1,578,765 in value) are sales. Our summary statistics are not far from the ones in the extant literature.

Both insider purchases and sales remain steady throughout our sample period, and the firm sizes are similar for the purchases and sales. It exhibits the broadly consistent occurrence of purchases and sales transaction. In evaluating the costs and implications of insider trading, the relative volume is of greatest significance. We plot the monthly volume of insider transactions as a percentage of monthly FTSE-350 turnovers, with the volume of insider sales averaging 0.0262% of monthly FTSE-350 turnover and the insider sales accounting for 0.0822%. The upward sloping linear trend-lines display the proportionate growth of insider purchases and sales across the 10-year period examined.

## Empirical Results: Short-Term Cumulative Abnormal Returns (CAR)

We can draw a figure of the abnormal returns from insider trades in which we can see that insiders pursue a contrarian strategy, with prices increasing (decreasing) before sales (purchases) over the 20 days. However, the magnitude is much smaller than the one documented in the extant literature, with purchase transactions down −0.839% (Table 1) and pre-trade and sale transactions up 0.755% pre-trade (Table 1). These results are vs. 2.85 and 5.97%, respectively in Friederich et al. (2002), −4.77 and 2.50% in Hillier and Marshall (2002), as well as −2.01 and 2.29% in Fidrmuc et al. (2006, Table 3 on p. 2590). Regarding post-transaction returns, purchases in Panel A in Table 1 yield negative abnormal returns (significant according to the BMP test and insignificant according to other tests), indicating no profitability in this case. The market model adjusted post-sales returns are negative and significant to the tune of −0.752% (Table 1), which is again much smaller than its counterparts, such as −1.46% in Friederich et al. (2002) and −1.37% in Hillier and Marshall (2002). Our conclusion holds if we compare 1-day and 4-day post-transaction returns with Fidrmuc et al. (2006) and other uncited studies, whether we focus on purchases or sales. These profits can hardly compensate an average roundtrip trading cost of 2.9% for FTSE-350 (Ellis and Thomas, 2004), while latter studies typically report a higher trading cost: 6.2% for all UK stocks in Kassimatis (2011) or 8.1% (11.3%) for stocks with high (low) accruals in Soares and Stark (2009), among others.

**Table 1:** Short-term abnormal returns.

**Days**	**% AR**	**%CAR t_**−20, *t*+20**_**	**%CAR t_**−20, *t*0**_**	**%CAR _***t*0, *t*+20**_**
**Purchases**				
−20	−0.062	−0.062	−0.062	
−15	−0.07	−0.201	−0.201	
−10	−0.034	−0.355	−0.355	
−5	−0.064	−0.588	−0.588	
−4	−0.063	−0.651	−0.651	
−3	−0.03	−0.681	−0.681	
−2	−0.044	−0.725	−0.725	
−1	−0.112	−0.837	−0.837	
0	−0.002	−0.839	−0.839	0.000
1	0.027	−0.812		0.027
2	0.055	−0.757		0.082
3	0.034	−0.723		0.116
4	−0.007	−0.73		0.109
5	−0.04	−0.77		0.069
10	−0.015	−0.86		−0.021
15	−0.008	−0.89		−0.05*o*
20	0.002	−0.913		−0.074
*Cumul t*	−	−14.1497**	−17.8698**	−1.7672
*t–BMP*	−	−17.7844**	−19.1078**	−4.8547**
*t–Corrado*	−	−5.8757**	−6.1091**	−1.4353
**Sales**				
−20	0.03	0.03	0.03	
−15	0.073	0.288	0.288	
−10	0.029	0.57	0.57	
−5	0.039	0.705	0.705	
−4	0.109	0.814	0.814	
−3	0.099	0.913	0.913	
−2	0.021	0.934	0.934	
−1	0.037	0.971	0.971	
0	−0.216	0.755	0.755	0.000
1	−0.049	0.706		−0.049
2	−0.011	0.695		−0.06
3	−0.059	0.636		−0.119
4	−0.039	0.597		−0.158
5	−0.005	0.591		−0.163
10	−0.02	0.408		−0.347
15	−0.029	0.21		−0.545
20	−0.039	0.003		−0.752
*Cumul t*	−	0.025	8.3295**	−11.72**
*t–BMP*	−	−0.4774	7.7848**	−11.9791**
*t–Corrado*	−	2.0714^*^	4.8807**	−3.1663**

*The table reports the short-term average Abnormal Returns (AR), short-term average Cumulative Abnormal Returns (CAR) on selected days around insider trading in the 1st and 2nd column, as well as the short-term average CAR from the beginning of the event window and from the day of the trade in the 3rd and 4th column, respectively. Cumul t denotes the cumulative t-test statistic calculated as in Brown and Warner (1985, p. 29), while t-BMP and t-Corrado denote the cumulative (Boehmer et al., 1991) statistic as in Equation (3) and the cumulative (Corrado, 1989) statistic as in Equation (4), respectively*.

* (**) indicates statistical significance at the 5% (1%) level.

## Robustness

Seeking to add robustness to our main findings above, we redesign our research strategy. First, we consider alternative window lengths from 1 to 120 days and report the results in Table 2. Second, we follow Friederich et al. (2002) and Fidrmuc et al. (2006) to take into account the thin trading (stale quotes) and report the results in Table 3. Third, we consider alternative benchmark models other than the simple one-factor market model, including the Fama-French three-factor model as well as the Fama-French-Carhart four-factor models. Fourth, we use the calendar-time portfolio approach instead of the standard event approach listed in Section Methodology and Data. Fifth, we use a value-weighted portfolio approach rather than the equal-weighted portfolio approach above. Sixth, we match our sample with the extant studies using data before the 2000s. Seventh, we break our sample into quantiles by size and value to check whether our conclusion is an artifact of the size or value anomaly and report the results in Table 4. Eighth, instead of using the market model to obtain risk-adjusted abnormal returns, we obtain non-risk-adjusted abnormal return by arbitrarily imposing the constraints that alphas equal to zero and betas equal to one. Ninth, we exclude the period of the global financial crisis (2008–09) to check whether our results are driven by the crisis. The results are reminiscent of our main findings above; therefore, we omit most of them for brevity.

**Table 2:** Medium-term abnormal returns.

**Days**	**%AR**	**%*CAR*1_*t*−120, *t*+120_**	**%*CAR*_*t*−120, *t*0_**	**%*CAR*_*t*0, *t*+120_**
**Purchases**				
−120	0	0		
−100	−0.01	−0.18	−0.18	
−80	0	−0.3	−0.3	
−40	−0.04	−0.66	−0.66	
−20	−0.06	−1.19	−1.19	
0	−0.01	−2.07	−2.07	0.00
20	0.00	−2.24		−0.17
40	−0.03	−2.57		−0.49
80	−0.03	−2.97		−0.9
100	−0.01	−3.14		−1.06
120	0.01	−3.38		−1.31
*Cumul t*	−	−24.2123^**^	−20.9373^**^	−13.2984^**^
*t-BMP*	−	−22.811^**^	−22.5505^**^	−13.4494^**^
**Sales**				
−120	−0.05	−0.05	−0.05	
−100	0.00	−0.46	−0.46	
−80	0.08	−0.53	−0.53	
−40	0.01	0.22	0.22	
−20	0.03	0.84	0.84	
0	−0.21	1.61	1.61	0
20	−0.04	0.87		−0.74
40	−0.03	0.22		−1.39
80	0.02	−1.41		−3.02
100	−0.02	−2.22		−3.82
120	−0.09	−2.79		−4.4
*Cumul t*		−5.82^**^	5.6835^**^	−15.5062^**^
*t–BMP*	−	−4.3941^**^	5.3019^**^	−14.582^**^

*The table reports the medium-term average Abnormal Returns (AR), medium-term average Cumulative Abnormal Returns (CAR) on selected days around insider trading in the 1st and 2nd column, as well as the medium-term average CAR from the beginning of the event window and from the day of the trade in the 3rd and 4th column, respectively. Cumul t denotes the cumulative t-test statistic calculated as in Brown and Warner (1985, p. 29), while t-BMP and t-Corrado denote the cumulative (Boehmer et al., 1991) statistic as in Equation (3) and the cumulative (Corrado, 1989) statistic as in Equation (4), respectively*.

** * indicates statistical significance at the 1% level*.

**Table 3:** CAR with Scholes and Williams (1977) thin trading adjustment.

**Sample**	**%*CAR*_*t*−20, *t*0_**	**%*CAR*_*t*0, *t*+20_**	***CAR*_*t*−120, *t*0_**	**%*CAR*_*t*0, *t*+120_**
**Purchases**				
	−0.83	−0.11	−2.18	−1.41
*Cumul t*	−18.5673**	−2.7144**	−16.9058**	−11.6874**
*t*–BMP	−18.3412**	−4.7651**	−23.95**	−13.3892^*^
*t*–Corrado	−5.7611**	−1.5217	−	−
**Sales**				
	0.72	−0.96	1.81	−4.29
*Cumul t*	7.9246**	−11.7257**	6.6294**	−14.7659**
*t*–BMP	7.5321**	−11.8227**	7.0194**	−15.8541**
*t*–Corrado	4.5138**	−3.1936**	−	−

*The table reports the short-term average Cumulative Abnormal Returns (CAR) from the beginning of the event window and from the day of the trade in the 1st and 2nd column, as well as the medium-term average CAR from the beginning of the event window and from the day of the trade in the 3rd and 4th column, respectively. Cumul t denotes the cumulative t-test statistic calculated as in Brown and Warner (1985, p. 29), while t-BMP and t-Corrado denote the cumulative (Boehmer et al., 1991) statistic as in Equation (3) and the cumulative (Corrado, 1989) statistic as in Equation (4), respectively*.

* (**) indicates statistical significance at the 5% (1%) level.

**Table 4:** Size and value composition.

**Summary statistics**	**Net purchase ratio**	**Net number ratio**	**Net value ratio**
**Size quintiles**			
Q1–Large	0.7123	−0.3774	−0.4814
Q2	0.6182	−0.2813	−0.5086
Q3	0.5986	−0.5886	−0.5329
Q4	0.6673	−0.4859	−0.6949
Q5–Small	0.7817	0.1875	−0.2592
**Value quintiles**			
Q1–Growth	0.6485	−0.6583	−0.7476
Q2	0.6118	−0.7829	−0.7133
Q3	0.6531	−0.2957	−0.4219
Q4	0.7061	−0.3408	−0.3955
Q5–Value	0.8053	0.3014	−0.1632

*The table reports the summary statistics of the size and value quintiles, in which FTSE350 constituents are ranked by market capitalization and market-to-book ratio as of 1st January each year. Annual FTSE350 size and market-to-book percentiles are obtained and insider transactions are ranked into corresponding quintiles relative to such. The Net Purchase Ratio is the gross number of purchase transactions over the gross number of sale transactions, while the Net Number Ratio (Net Value Ratio) is the gross number (value) of shares purchased over the gross number (value) of shares sold*.

## Concluding Remarks

Finance (and to a lesser extent, Economics) is the last field to take replications of published results seriously, although replication studies in other scientific fields routinely appear in top journals such as *Nature* and *Science* (see Harvey et al., 2016; Harvey, 2017; Hou et al., 2017 and the references therein). This study complements several recent studies that doubt the credibility of the anomalies against the weak-form and semi-strong-form EMH. For the first time, we have questioned, replicated, examined, and re-evaluated the insider trading anomaly, which is against the strong-form EMH.

Using detailed insider trading data on the UK stock market over the last decade, we find much lower abnormal returns from insider trading than documented in the literature and the associated trading costs, which suggests that the market efficiency of individual stocks may depend on their sizes from a non-US perspective.

Although the literature has described the small-firm effect, our contribution illustrates that because the small-firm effect is so large, even the strong-form of EMH holds to a larger extent than previously recognized, after excluding the small firms. While the UK insider trading regulation system displays certain idiosyncrasies, we expect similar results for other insider trading regulation systems.

## Data Availability Statement

Publicly available datasets were analyzed in this study. This data can be found here: Morningstar Premium Director Share Dealings Database, https://www.morningstar.co.uk.

## Author Contributions

JM: writing draft. LH: editing. ZL: proofreading. All authors contributed to the article and approved the submitted version.

## Funding

This study received research support by Guangdong Province's Philosophy and Social Sciences Thirteenth Five-Year Plan Project (GD19CJY22).

## Conflict of Interest

The authors declare that the research was conducted in the absence of any commercial or financial relationships that could be construed as a potential conflict of interest.

## Publisher's Note

All claims expressed in this article are solely those of the authors and do not necessarily represent those of their affiliated organizations, or those of the publisher, the editors and the reviewers. Any product that may be evaluated in this article, or claim that may be made by its manufacturer, is not guaranteed or endorsed by the publisher.

## Footnotes

^1^Interestingly, Friederich et al. (2002) use FTSE-All-share excluding FTSE100 with a simple market model.

^2^FTSE Russell Factsheet (2017). Available online at: http://www.ftse.com/analytics/factsheets/home/search

## References

[B1] Boehmer E. Musumeci J. Poulsen A. B. (1991). Event-study methodology under conditions of event-induced variance. J. Fin. Econ. 30, 253–272. 10.1016/0304-405X(91)90032-F

[B2] Brown S. J. Warner J. B. (1985). Using daily stock returns: the case of event studies. J. Fin. Econ. 14, 3–31. 10.1016/0304-405X(85)90042-X

[B3] Campbell C. J. Wesley C. E. (1993). Measuring security price performance using daily NASDAQ returns. J. Fin. Econ. 33, 73–92. 10.1016/0304-405X(93)90025-7

[B4] Corrado C. J. (1989). A nonparametric test for abnormal security-price performance in event studies. J. Fin. Econ. 23, 385–395. 10.1016/0304-405X(89)90064-0

[B5] Ellis M. Thomas D. C. (2004). Momentum and the FTSE 350. J. Asset Manage. 5, 25–36. 10.1057/palgrave.jam.2240125

[B6] Fama E. (1998). Market efficiency, long-term returns, and behavioral finance. J. Fin. Econ. 49, 283–306. 10.1016/S0304-405X(98)00026-9

[B7] Fama E. F. French K. R. (2008). Dissecting anomalies. J. Fin. 63, 1653–1678. 10.1111/j.1540-6261.2008.01371.x

[B8] Fidrmuc J. P. Goergen M. Renneboog L. (2006). Insider trading, news releases, and ownership concentration. J. Fin. 61, 2931–2973. 10.1111/j.1540-6261.2006.01008.x

[B9] Friederich S. Gregory A. Matatko J. Tonks I. (2002). Short-run returns on around the trades of corporate insiders on the LSE. Eur. Fin. Manage. 8, 69–87. 10.1111/1468-036X.00174

[B10] Harvey C. R. (2017). Presidential Address: the scientific outlook in financial economics. J. Fin. 72, 1399–1440. 10.1111/jofi.12530

[B11] Harvey C. R. Liu Y. Zhu H. (2016). … and the cross-section of expected returns. Rev. Fin. Stud. 29, 5–68. 10.1093/rfs/hhv059

[B12] Hillier D. Marshall A. P. (2002). The market evaluation of information in directors' trades. J. Bus. Fin. Account. 29, 77–110. 10.1111/1468-5957.00424_29_1-2

[B13] Hou K. Xue C. Zhang L. (2017). Replicating Anomalies. Fisher College of Business Working Paper No. 2017-03-010. Available online at: https://ssrn.com/abstract=2961979

[B14] Kassimatis K. (2011). Risk aversion with local risk seeking and stock returns: evidence from the UK market. J. Bus. Fin. Account. 38, 713–739. 10.1111/j.1468-5957.2011.02243.x

[B15] Scholes M. Williams J. (1977). Estimating betas from non-synchronous data. J. Financ. Econ. 5, 308–328. 10.1016/0304-405X(77)90041-1

[B16] Soares N. Stark A. W. (2009). The accruals anomaly-can implementable portfolio strategies be developed that are profitable net of transactions costs in the UK? Account. Bus. Res. 39, 321–345. 10.1080/00014788.2009.9663371

